# Genome-wide identification *AINTEGUMENTA-like* (*AIL*) genes in *Brassica* species and expression patterns during reproductive development in *Brassica napus* L.

**DOI:** 10.1371/journal.pone.0234411

**Published:** 2020-06-08

**Authors:** Shulin Shen, Fujun Sun, Meichen Zhu, Si Chen, Mingwei Guan, Rui Chen, Fang Tang, Nengwen Yin, Xinfu Xu, Zhanglin Tang, Jiana Li, Kun Lu, Cunmin Qu

**Affiliations:** 1 Chongqing Rapeseed Engineering Research Center, College of Agronomy and Biotechnology, Southwest University, Beibei, Chongqing, China; 2 Academy of Agricultural Sciences, Southwest University, Chongqing, China; Huazhong University of Science and Technology, CHINA

## Abstract

The AINTEGUMENTA-like (AIL) proteins, which belong to the AP2 family, play important roles in regulating the growth and development of plant organs. The AIL family has not yet been comprehensively studied in rapeseed (*Brassica napus*), an allotetraploid and model organism for the study of polyploid evolution. In the present study, 99 *AIL* family genes were identified and characterized from *B*. *rapa*, *B*. *oleracea*, *B*. *napus*, *B*. *juncea*, and *B*. *nigra* using a comprehensive genome-wide study, including analyses of phylogeny, gene structure, chromosomal localization, and expression pattern. Using a phylogenetic analysis, the *AIL* genes were divided into eight groups, which were closely related to the eight *AtAIL* genes, and which shared highly conserved structural features within the same subfamily. The non-synonymous/synonymous substitution ratios of the paralogs and orthologs were less than 1, suggesting that the *AIL* genes mainly experienced purifying selection during evolution. In addition, the RNA sequencing data and qRT-PCR analysis revealed that the *B*. *napus AIL* genes exhibited organ- and developmental stage-specific expression patterns. Certain genes were highly expressed in the developing seeds (*BnaAIL1*, *BnaAIL2*, *BnaAIL5*, and *BnaAIL6*), the roots (*BnaANT*, *BnaAIL5*, and *BnaAIL6*), and the stem (*BnaAIL7B*). Our results provide valuable information for further functional analysis of the *AIL* family in *B*. *napus* and related *Brassica* species.

## Introduction

Plant growth and developmental processes are influenced by the complex external environment and internal developmental factors [1, 2]. Deciphering the molecular networks contributing to plant growth and development is therefore an important research goal. Over the past few decades, many internal regulatory factors contributing to plant growth and development have been described, including *AINTEGUMENTA* (*ANT*) [1, 3–6], *ANT-LIKE*/*PLETHORA* (*AIL*/*PLT*) [4, 7, 8], *AUXIN-REGULATED GENE INVOLVED IN ORGAN SIZE* (*ARGOS*) [9, 10], and various growth-regulating factors (*GRF*s) [11–14]. Among them, the *AIL* genes comprise an APETALA 2 (AP2) subfamily known to be involved in growth‐related processes in a variety of plants [3, 4, 15–18]. These genes play an important role in regulating the growth and development of organs (e.g., embryos and flowers) [1, 3, 19–22]; for example, loss-of-function mutations in *ANT* resulted in *A*. *thaliana* with smaller organs [18, 21, 23], while overexpression of the *ANT* genes caused increases in organ size [1, 24, 25]. In addition, some *AIL* genes were shown to be involved in the differentiation of embryogenic stem cells from somatic cells in *A*. *thaliana* [26–28], oil palm (*Elaeis guineensis*) [29], and coconut (*Cocos nucifera*) [30, 31]. The ectopic expression of *EgAP2-1* alters leaf morphology and enhances the regeneration capacity of the oil palm [29], while coconut nucellar development is also regulated by *AIL* genes [30]. Transgenic *Arabidopsis* plants expressing *AtAIL6* exhibited changes in floral organ size and morphology associated with alterations in the pattern and duration of cell divisions within the developing organs, while the *ant ail6* double mutant displayed a premature differentiation of their floral meristem cells [28]. Numerous studies have also revealed that the *AIL* family genes are involved in root development and abiotic stress responses, including *AIL6/PLETHORA3* (*PLT3*), *PLT1*, *PLT2*, and *BABY BOOM* (*BBM*) [17, 32]. As the excellent evolutionary model to investigate the expansion of gene families [33], the *AIL* family members have not been well studied in the *Brassica* genus.

In the present study, we investigated the *AIL* family members in various *Brassica* species (*B*. *rapa*, *B*. *oleracea*, *B*. *napus*, *B*. *juncea*, and *B*. *nigra*) using a genome-wide bioinformatics analysis, exploring their exon-intron organization, motif compositions, gene duplications, chromosome distribution, phylogeny, and synteny. We also examined the expression patterns of selected *B*. *napus AIL* genes in different tissues. Based on these data, the functions of the *AIL* genes in *B*. *napus* were predicted, providing a reference for the functional verification and utilization of the *AIL* family in *B*. *napus* and other *Brassica* species.

## Materials & methods

### Identification and characterization of AIL family proteins

The amino acid sequences of the AIL proteins were obtained from The Arabidopsis Information Resource (TAIR10) database (ftp://ftp.arabidopsis.org) and used as queries for a BLASTp search of the whole *Brassica* genome sequences stored in the *Brassica* database [34], including the *B*. *rapa* genome V3.0, *B*. *oleracea* genome V1.1, *B*. *napus* genome V5, *B*. *juncea* genome V1.5, and *B*. *nigra* genome V1.1 (http://brassicadb.org/brad/index.php), respectively. Candidate sequences with E-values ≤ 1 × 10^−10^ were identified and confirmed using the NCBI CD Searches-Tool (https://www.ncbi.nlm.nih.gov/Structure/bwrpsb/bwrpsb.cgi). A BLAST search of the *Brassica* protein database was performed to search for the *AIL* genomic sequences using the NCBI blast+ software (ftp://ftp.ncbi.nlm.nih.gov/blast/executables/blast+/LATEST/). The coding sequences (CDS) of the *AILs* were identified using BLASTn searches of the *Brassica* genome database. The candidate proteins were named using the species abbreviation of the source organism (italicized), the name of the clade determined in the subsequent phylogenetic analysis (below), and the position in the clade (e.g., *BolAIL1A* and *BnaAIL1A*). The physicochemical properties of each deduced protein, including the molecular weight (MW), isoelectric point (pI), and the grand average of hydropathy (GRAVY) value, were determined using the online ExPASy-ProtParam tool (http://web.expasy.org/protparam/).

### Multiple sequence alignment and phylogenetic analysis of the *AIL* family

The deduced amino acid sequences of the AIL proteins from *A*. *thaliana* and various *Brassica* species, including *B*. *rapa*, *B*. *oleracea*, *B*. *napus*, *B*. *juncea*, and *B*. *nigra*, were subjected to multiple protein sequence alignments using the ClustalW software (Version 2.0) with default settings [35]. To illustrate the evolutionary relationships of the AILs in the *Brassica* genus, a neighbor-joining (NJ) phylogenetic tree was generated using the MEGA v6.0 program (Tokyo Metropolitan University, Tokyo, Japan) with the best-fit model, a Jones-Taylor-Thornton (JTT) matrix and incorporates a proportion of invariant sites (+I) and the gamma distribution for modeling rate heterogeneity (+G). We performed NJ analysis in MEGA v6.0 with bootstrap test with 1,000 replicates [36]. The phylogenetic trees were visualized using evolview v3 (https://www.evolgenius.info/evolview/) [37].

### Conserved motif recognition and gene structure analysis

The exon-intron structures of the *AIL* family genes were analyzed using the TBtools software (https://github.com/CJ-Chen/TBtools). Conserved motifs were identified using Multiple Expectation Maximization for Motif Elucidation (MEME v4.12.0, http://meme-suite.org/tools/meme) with the following parameters: number of repetitions, any; maximum number of motifs, 10; and optimum width of each motif, between 6 and 300 residues [38].

### Chromosomal distribution and gene duplication

All the identified *AIL* family genes were mapped to their respective chromosomes based on the physical location information from the *Brassica* species genome database using MapChart v2.0 (https://www.wur.nl/en/show/Mapchart.htm) [39]. A Multiple Collinearity Scan toolkit (MCScanX) was adopted to analyze the gene synteny events, using the default parameters [40]. To examine the syntenic relationships of the orthologous *AIL* family genes obtained from *B*. *napus* and other selected species, synteny maps were constructed using the Circos software [41]. Non-synonymous (ka) and synonymous (ks) substitutions for each of the duplicated *AIL* family genes were calculated using KaKs_Calculator v2.0 [42].

### Total RNA extraction and qRT-PCR analysis

Total RNA was isolated from the samples using a DNA away RNA Mini-Prep Kit (Sangon Biotech, Shanghai, China). For the tissue-specific expression analysis, RNA was extracted from the roots, stems, leaves, buds, flowers, and seeds, and pretreated with gDNA Eraser (Takara Bio, Kusatsu, Japan). Subsequently, 1 μg total RNA was used to synthesize the first-strand cDNA using an RNA PCR Kit (AMV) v3.0 (Takara Bio). The cDNA was subjected to a RT-qPCR analysis using SYBR Premix Ex Taq II (Takara Bio) on a Bio-Rad CFX96 Real-Time System (Bio-Rad Laboratories, Hercules, CA, USA), as previously described [43]. *BnACTIN7* (EV116054) was used as a reference gene to normalize the *AIL* gene expression levels via the 2^−ΔΔCt^ method [44]. Three technical replicates were performed for all experiments. The specific primer sequences used in this study were obtained from the qPCR Primer Database [45] and are listed in S1 Table.

## Results

### Identification and characterization of the *AIL* family genes

Eight *A*. *thaliana* AIL family protein sequences were acquired from the TAIR10 database and used as queries to identify the AIL family in various *Brassica* species (*B*. *rapa*, *B*. *oleracea*, *B*. *napus*, *B*. *juncea*, and *B*. *nigra*) using the BLASTp program. In total, 99 AIL family proteins were identified in these species, including 26 in *B*. *juncea*, 29 in *B*. *napus*, 15 in *B*. *oleracea*, 14 in *B*. *nigra*, and 15 in *B*. *rapa* (Table 1 and S2 Table). More AIL family proteins were identified in the *Brassica* species than in *A*. *thaliana*. Based on their homology with the corresponding *A*. *thaliana AIL* family genes, the identified *Brassica AIL* family genes were named *ANT* or *AIL1–7* (Table 1 and S2 Table). A species-specific prefix was included, while a capital letter suffix was used to represent the gene number within each clade.

**Table 1:** Statistics of *AIL* family genes between *A*. *thaliana* and five *Brassica* species.

Gnen Family	*A*. *thaliana*	*B*. *rapa*	*B*. *oleracea*	*B*. *nigra*	*B*. *juncea*	*B*. *napus*
*ANT*	1	3	3	3	5	5
*AIL1*	1	1	1	0	1	3
*AIL2*	1	2	2	2	4	2
*AIL3*	1	2	2	2	4	5
*AIL4*	1	2	2	2	4	3
*AIL5*	1	1	1	1	2	2
*AIL6*	1	3	3	3	5	6
*AIL7*	1	1	1	1	2	2
Total	8	15	15	14	26	29

The lengths of the AIL protein sequences ranged from 290 (BnaAIL1A) to 652 (BjuANTD) amino acids were almost distributed across the whole chromosomes; the highest content was on chromosome BniB02, including 5 *AIL* genes. The MW varied from 32.52 (BnaAIL1A) to 71.78 kDa (BjuANTD), and the pIs ranged from 5.47 (BjuAIL3A) to 9.56 (BnaANTB and BniANTA), with 23 pIs > 7 and the remaining pIs ≤ 7 (S2 Table).

### Phylogenetic and classification analysis of the AIL proteins

**Fig 1:**
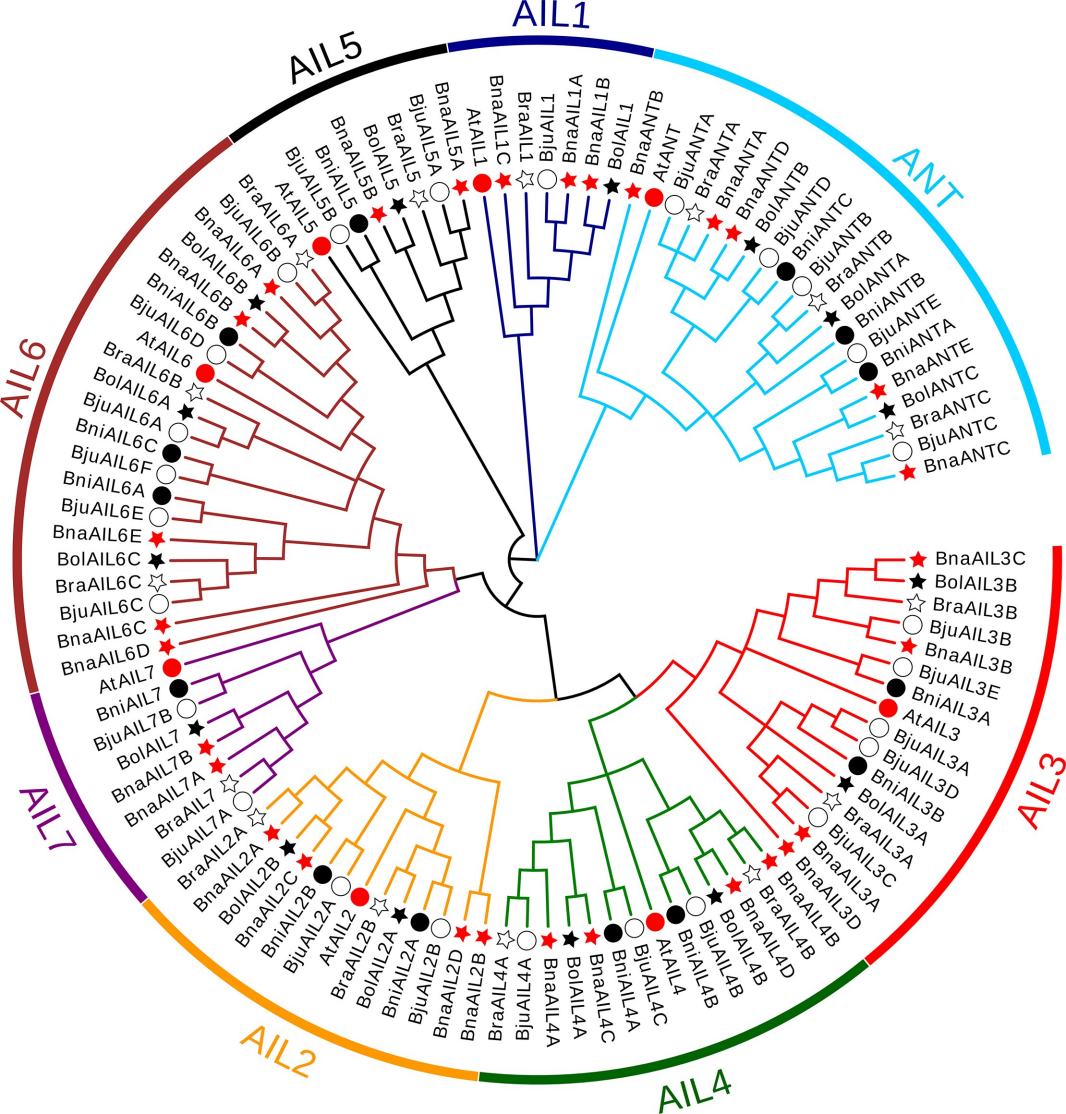
Neighbor-Joining (NJ) phylogenetic tree of the AIL family proteins in *A*. *thaliana* and various *Brassica* species. The AIL family was divided into eight clades (ANT and AIL1–7), which are indicated by different colors. The red, white, and black stars indicate *B*. *napus*, *B*. *rapa*, and *B*. *oleracea*, respectively. The red, white, and black circles indicate *A*. *thaliana*, *B*. *juncea*, and *B*. *nigra*, respectively.

To investigate the evolutionary relationships among the AIL family, the protein sequences of AILs fromthe *A*. *Thaliana* and various *Brassica* species were used to generate the phylogenetic tree in this study, we constructed a NJ phylogenetic tree using the *A*. *thaliana* AIL proteins as a reference. We showed that the 107 *A*. *thaliana* and *Brassica* AIL protein sequences were classified into eight clades: the ANT clade and clades AIL1 to AIL7 (Fig 1). However, no BniAIL1 homologs were found in *B*. *nigra*, and the AIL3 and AIL4 subgroups were located in the same phylogenetic branch, indicating that these genes are more closely related to each other than to the other clades. Their similarity may be related to their shared involvement in the development of the lateral root primordia [21]. In general, the AIL proteins in the allotetraploids (*B*. *napus* and *B*. *juncea*) and their diploid progenitors (*B*. *rapa*, *B*. *oleracea*, and *B*. *nigra*) were related to their corresponding *A*. *thaliana* homologs in each clade (Fig 1), suggesting that the AIL proteins among these species have close evolutionary relationships.

### Gene structure and conserved motif analysis of the *AIL* family genes

**Fig 2:**
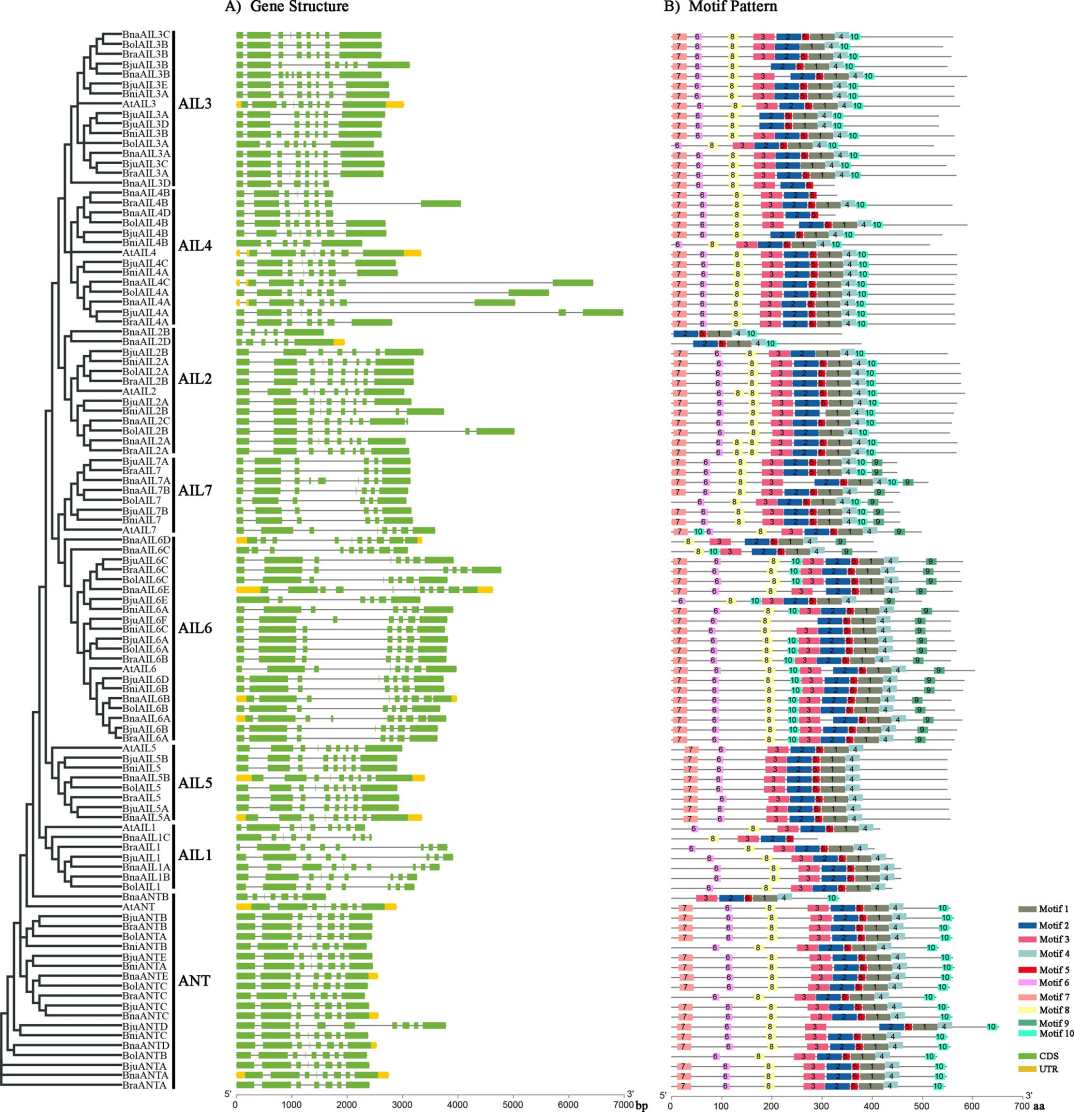
Characteristics of the identified AIL family genes and proteins in *A*. *thaliana* and selected *Brassica* species. **A)** Exons and introns are represented by green boxes and gray lines, respectively. **B)** The conserved motifs of the AIL proteins. A total of 10 motifs (number 1–10) were identified using MEME v4.12.0 (http://meme-suite.org/tools/meme), and are indicated as differently colored boxes. Yellow boxes represent upstream or downstream untranslated regions. Bra, *B*. *rapa*; Bol, *B*. *oleracea*; Bni, *B*. *nigra*; Bna, *B*. *napus*; Bju, *B*. *juncea*.

To further investigate the AIL proteins in each clade, their corresponding gene structures and conserved motifs were analyzed (Fig 2). Accordingly, the numbers of exons/introns within each *AIL* family clade were similar to each other, whether they originated from the allotetraploids (*B*. *napus* and *B*. *juncea*) or their diploid progenitors (*B*. *rapa*, *B*. *oleracea*, and *B*. *nigra*). A statistical analysis revealed that their numbers of exons typically ranged from six to nine (in 91% (97/107) of *AIL* family genes), indicating that the structures of the *AIL* family genes were conserved during polyploidization. *BnaAIL2B* contained the fewest exons (five), while the highest exon numbers were found in *BnaAIL1A* (10), *BnaAIL3B* (10), *BnaAIL6D* (10), and *BnaAIL6E* (11), respectively (Fig 2A and S2 Table). In general, the exon-intron patterns within the same phylogenetic classification group shared the high similarity between the *A*. *thaliana* and *Brassica* species (Fig 2A and S2 Table), indicating that they might be resulted by the replication of these sequences and supporting that the classification result is reliable.

In addition, the conserved motifs in the AIL proteins were predicted using MEME v4.12.0 (http://meme-suite.org/tools/meme). A total of ten conserved motifs were identified in the 107 AIL family members from *A*. *thaliana* and the selected *Brassica* species. The number of conserved motifs are different in each subgroup; for example, proteins in the AIL1 and AIL5 subfamilies contained eight motifs; those in the ANT, AIL2, AIL3, and AIL4 subfamilies had nine; and the AIL6 and AIL7 subfamilies contained ten (Fig 2B). Among these, the same conserved motifs were also widely observed in the paralogous/orthologous AIL family members; for instance, motifs 1 and 2 were found in every AIL family (Fig 2B), suggesting that they have a conserved position and functional similarity between *A*. *thaliana* and *Brassica* species. In addition, motif 9 was distributed in both the AIL6 and AIL7 subfamily, but motif 8 was not detected in the AIL5 subfamily (Fig 2B), indicating that these motifs were selectively distributed in certain AIL proteins. This specific distribution suggests that these motifs may have specific functions in the *A*. *thaliana* and *Brassica* AILs.

### Conserved amino acid sequences within the AP2 domain

**Fig 3:**
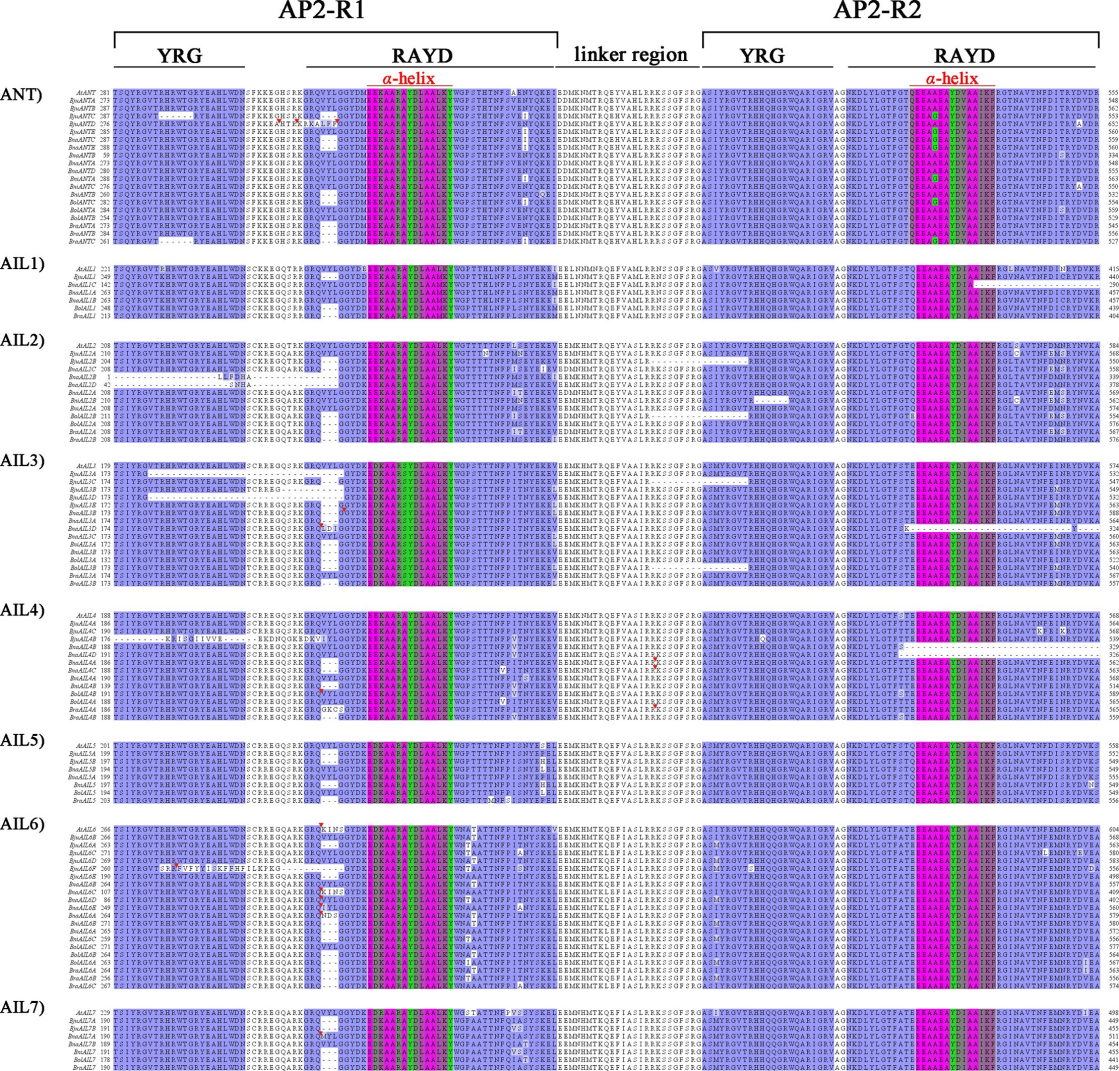
Alignment of the AIL protein sequences from *A*. *thaliana* and various *Brassica* species. Only the alignment sequences of the region from AP2-R1 to AP2-R2 are shown; detailed information is provided in S1 Fig. Blue shading represents identical conserved amino acid residues. Color shading represents an α-helix. The red triangles indicate locations at which part of a sequence was hidden for this alignment. A detailed description of the two AP2 domains is provided in S3 Table. Bra, *B*. *rapa*; Bol, *B*. *oleracea*; Bni, *B*. *nigra*; Bna, *B*. *napus*; Bju, *B*. *juncea*.

To investigate the sequences of the conserved AP2 domains in *A*. *thaliana* and the *Brassica* species, a multiple sequence alignment was performed using the 107 AIL proteins identified from *A*. *thaliana*, *B*. *rapa*, *B*. *oleracea*, *B*. *nigra*, *B*. *napus*, and *B*. *juncea* (Fig 3, S1 Fig and S3 Table). Two AP2 domains (AP2-R1 and AP2-R2) were located near the N- and C-terminal regions of the AIL proteins (Fig 3 and S1 Fig), which was consistent with previously published results [4, 6, 22]. These two AP2 domain regions were highly conserved in the AIL proteins. The lengths of the two AP2 domains were nearly constant between the AIL proteins, but varied in some of cases, such as for BnaAIL1C, BnaAIL2B, BnaAIL2D, BnaAIL3D, BjuAIL3A, BjuAIL3D, BjuAIL4B, BnaAIL4B, BnaAIL4D, and BjuAIL6F.

In addition, the AP2 domains all contained YRG and RAYD elements [46], the latter of which comprise a highly conserved 18-amino-acid core region predicted to form an amphipathic α-helix in the AP2 domains. The length of the RAYD α-helix was also highly conserved in most AP2 domains, except for BnaAIL1C, BnaAIL3D, BjuAIL4B, and BnaAIL4D (Fig 3 and S1 Fig). We found that the glycine residues within the RAYD element, which are involved in AP2 function [47], were identical in all the AP2-domain-containing proteins; therefore, the structure or function of the AP2 domains is likely to be associated with the invariant amino acid residues within the YRG and RAYD elements [46, 47]. Additionally, the sequences (25 aa) between the AP2-R1 and AP2-R2 domain were named as the linker regions with highly conserved, except in BjuAIL2B, BolAIL2B, BjuAIL3C, BolAIL3B, BnaAIL4A, BnaAIL4C, and BraAIL4A (Fig 3 and S1 Fig). A detailed description of the two AP2 domains is provided in S3 Table.

### Chromosomal localization analysis of the *AIL* genes among the *Brassica* species

**Fig 4:**
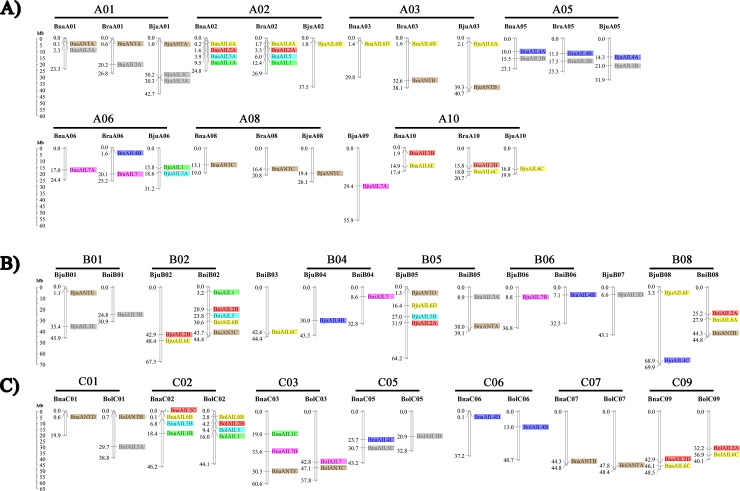
Chromosome distribution and analysis of the *AIL* family genes among the *Brassica* species. **A)** *AIL* genes distributed on the A subgenome, present in *B*. *rapa*, *B*. *napus*, and *B*. *juncea*. **B)** *AIL* genes distributed on the B subgenome, present in *B*. *juncea* and *B*. *nigra*. **C)** *AIL* genes distributed on the C subgenome, present in *B*. *oleracea* and *B*. *napus*. Genes from the same clades are represented by the same color (Fig 2). The labels on the corresponding chromosomes indicate the name of the source organism and the subgenome. The scales indicate the sizes of the various *Brassica* chromosomes (Mb). Bra, *B*. *rapa*; Bol, *B*. *oleracea*; Bni, *B*. *nigra*; Bna, *B*. *napus*; and Bju, *B*. *juncea*. Detailed information on the genes located on the scaffold sequences are not shown here.

The genome sequences of the allotetraploid species (*B*. *napus* and *B*. *juncea*) and their diploid progenitors (*B*. *rapa*, *B*. *oleracea*, and *B*. *nigra*) were acquired from the *Brassica* Database (http://brassicadb.org/brad/index.php), and the locations of the identified *AIL* family genes were drafted onto the corresponding chromosomes using Mapchart v2.0 software. As a result, 90 of the *AIL* family genes in the various *Brassica* plants could be mapped onto the A (39), B (26), and C (25) subgenomes, while nine were distributed onto different random chromosome and scaffold sequences that had not been assembled into the corresponding chromosomes (Fig 4, S2 Table). The *AIL* genes were unevenly distributed on the chromosomes, with between one and five genes on each. A comparison of the gene distributions of the allotetraploid species (*B*. *napus* and *B*. *juncea*) and their diploid progenitors (*B*. *rapa*, *B*. *oleracea*, and *B*. *nigra*) revealed the important result that many *AIL* family genes retained their relative positions in A_Bra_, A_Bju_, and A_Bna_; B_Bni_ and B_Bju_; and C_Bol_ and C_Bna_. For example, the *ANT* genes were located on chromosomes A01 and A08; the *AIL6* genes were present on chromosomes A02, A03, and A10; and the *AIL3* and *AIL4* were located on chromosome A05; the same patterns was also repeated on the B and C subgenomes (Fig 4B and 4C). This similarity suggests that these genes might have undergone whole-genome duplication events during the evolutionary process, and might have similar functions. In addition, some genes (e.g., *AIL1*, *AIL2*, and *AIL5* in A02; *ANT* in A03; and *AIL4* in A06) might have been lost during the evolution of *B*. *juncea* and *B*. *napus* due to the incomplete assembly of their chromosomes during their hybridization and polyploidization. Together, these results shed light on the evolutionary patterns in these subfamilies among related species.

### Synteny and duplicated gene analysis of the *AIL* family genes in *B*. *rapa*, *B*. *oleracea*, and *B*. *napus*

To investigate the patterns of retention or loss in the orthologous *AIL* family genes, we compared the relationships of the *AIL* genes between *A*. *thaliana* and *B*. *rapa*, *B*. *oleracea*, and *B*. *napus* (S4 Table and S2 Fig). Genes of the same clade were identified on many chromosomes (S2 Fig), suggesting that they were evolutionarily related and that most *AIL* genes were preserved during polyploidization.

**Fig 5:**
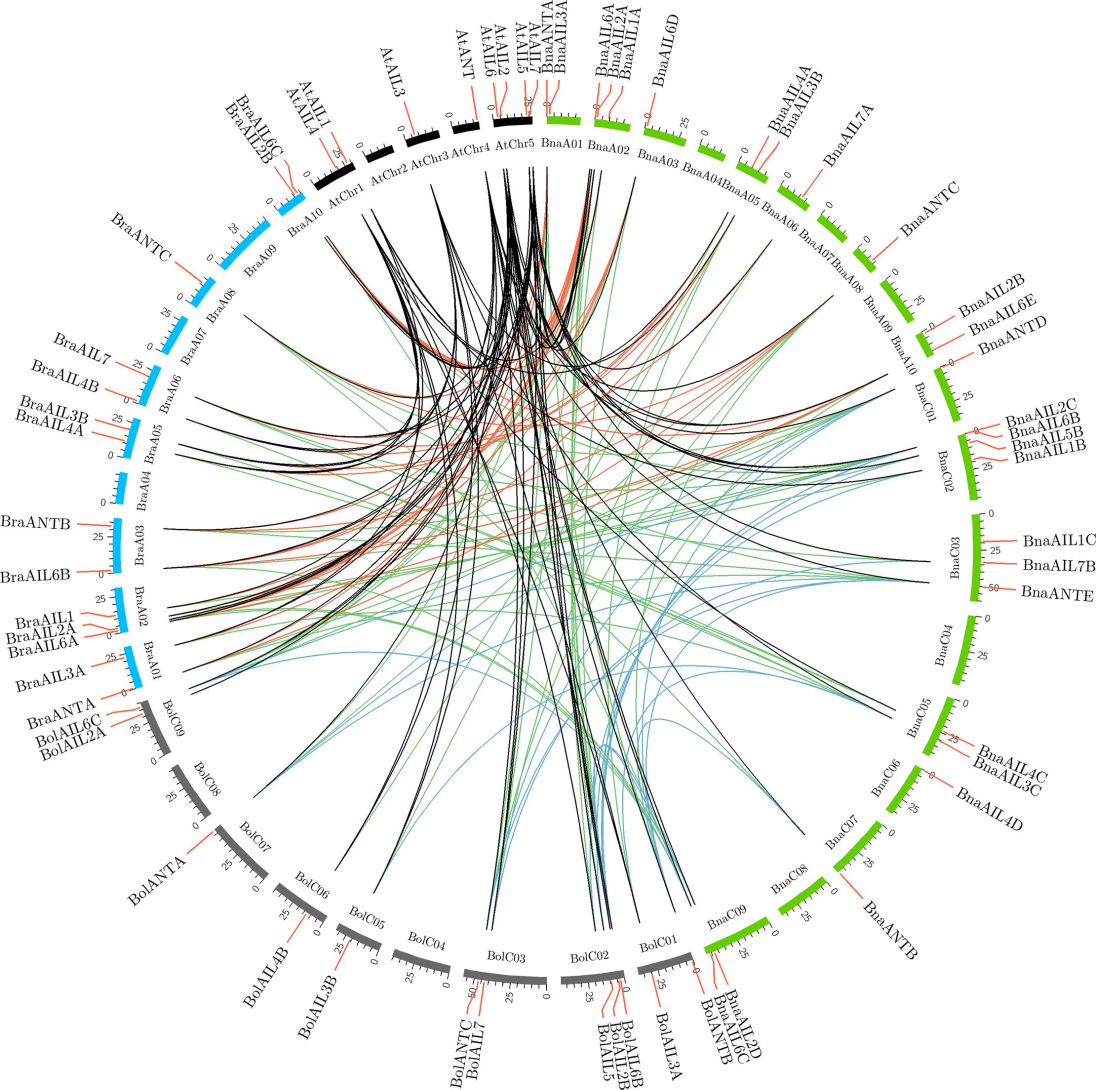
Genome-wide syntenic analysis of the *AIL* family genes among *A*. *thaliana*, *B*. *rapa*, *B*. *oleracea*, and *B*. *napus*. AtChr1 to AtChr5 are the five chromosomes in *A*. *thaliana*; BraA01 to BraA10 are the ten chromosomes in *B*. *rapa*; BolC01 to BolC09 are the nine chromosomes in *B*. *oleracea*; BnaA01 to BnaA10 and BnaC01 to BnaC09 represent the ten and nine chromosomes of the *B*. *napus* A and C subgenomes, respectively. The different colored lines represent the syntenic regions in *A*. *thaliana*, *B*. *rapa*, *B*. *oleracea*, and *B*. *napus*.

In addition, we compared the syntenic relationship of the *AIL* genes in *A*. *thaliana*, the allotetraploid *B*. *napus* (A_Bna_ and C_Bna_) and its diploid progenitors *B*. *rapa* (A_Bra_) and *B*. *oleracea* (C_Bol_), according to their corresponding syntenic information obtained from the BRAD database. A total of 15 *BraAIL* genes and 13 *BolAIL* genes showed a syntenic relationship with the eight *AtAIL* genes and 22 *BnaAIL* genes (Fig 5, S4 Table). Furthermore, the numbers of orthologous pairs identified in the comparisons of *AtAIL* and *BraAIL*, *AtAIL* and *BolAIL*, *AtAIL* and *BnaAIL*, *BraAIL* and *BnaAIL*, and *BolAIL* and *BnaAIL* were 23, 19, 26, 39, and 48, respectively. These results showed that the syntenic *AIL* gene pairs were widely distributed on the genomes of the allotetraploid (*B*. *napus*) and its diploid progenitors (*B*. *rapa* and *B*. *oleracea*).

We also calculated the nonsynonymous substitutions (Ka), synonymous substitutions (Ks), and Ka/Ks ratios of the *AIL* gene pairs to identify the evolutionary constraints acting on the *AIL* gene pairs, revealing that the Ka/Ks values of all orthologous *AIL* gene pairs were less than 1 (S4 Table). This suggests that the *AIL* family genes in *B*. *napus* and its diploid progenitors might have experienced strong purifying selective pressure after the duplication events.

### Expression profiles of the *BnaAIL* family genes in various *B*. *napus* organs

**Fig 6:**
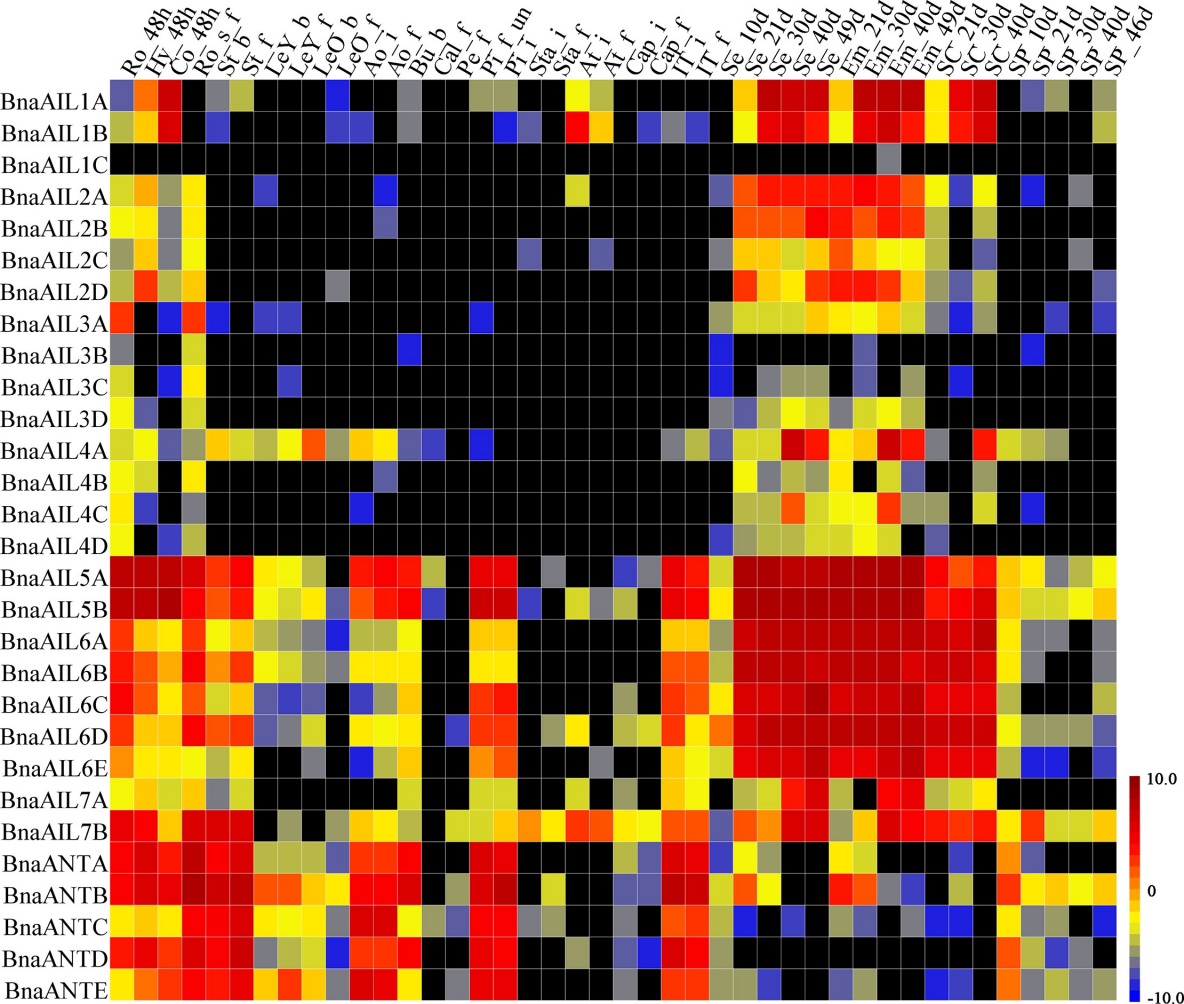
Expression profiles of the *BnaAIL* family genes in different tissues and organs. The abbreviations above the heatmap represent the different tissues and organs/developmental stages of *B*. *napus* ZS11, and are listed in S5 Table. The bar represents the log_2_ expression levels (FPKM). Black boxes indicate that no expression was detected in an RNA-seq analysis.

To investigate the putative functions of the *BnaAIL* family genes in regulating the growth and development of *B*. *napus*, we characterized the expression profiles of the *BnaAIL* genes in different tissues. This was achieved using the transcriptome sequencing datasets of *B*. *napus* ZS11 stored in National Genomics Data Center (BioProject ID PRJNA358784), which covered all stages of *B*. *napus* development and a variety of organs, including the roots, hypocotyl, cotyledon, stems, leaves, anthocaulus, buds, calyx, petals, pistil, stamens, anthers, capillament, initial apex, seeds, embryo, seed coat, and silique pericarp (Fig 6 and S5 Table). The expression profiles of these *AIL* family genes showed clear differences among these tissues, except for *BnaAIL1C* and *BnaAIL3B* that were not highly expressed in any of the tissues, suggesting that the genes of this family might perform a variety of biological functions in different tissues.

Furthermore, the members of specific *AIL* family clades displayed similar characteristics; for example, *BnaAIL1* to *BnaAIL4* were generally only expressed in the roots, hypocotyl, cotyledon, developing seed, embryo, and seed coat. The *BnaAIL5*, *BnaAIL6*, *BnaAIL7*, and *BnaANT* clades were widely expressed in all tissues, especially in the younger tissues; for example, the *BnaAIL5* and *BnaAIL6* family members showed higher expression levels in the developing seed, embryo, and seed coat. *BnaAIL5*, *BnaAIL7*, and *BnaANT* members were also expressed in the roots, hypocotyl, cotyledon, and stem, with *BnaAIL5* and *BnaANT* also being expressed in the anthocaulus. *BnaAIL5*, *BnaAIL6*, and *BnaANT* were particularly highly expressed in the pistil and, in addition to *BnaAIL7*, in the initial apex. Our results suggest that the *AIL* family genes play important roles in the processes of growth and development in *B*. *napus*.

### Expression patterns of the *BnaAIL* genes revealed using qRT-PCR analysis

**Fig 7:**
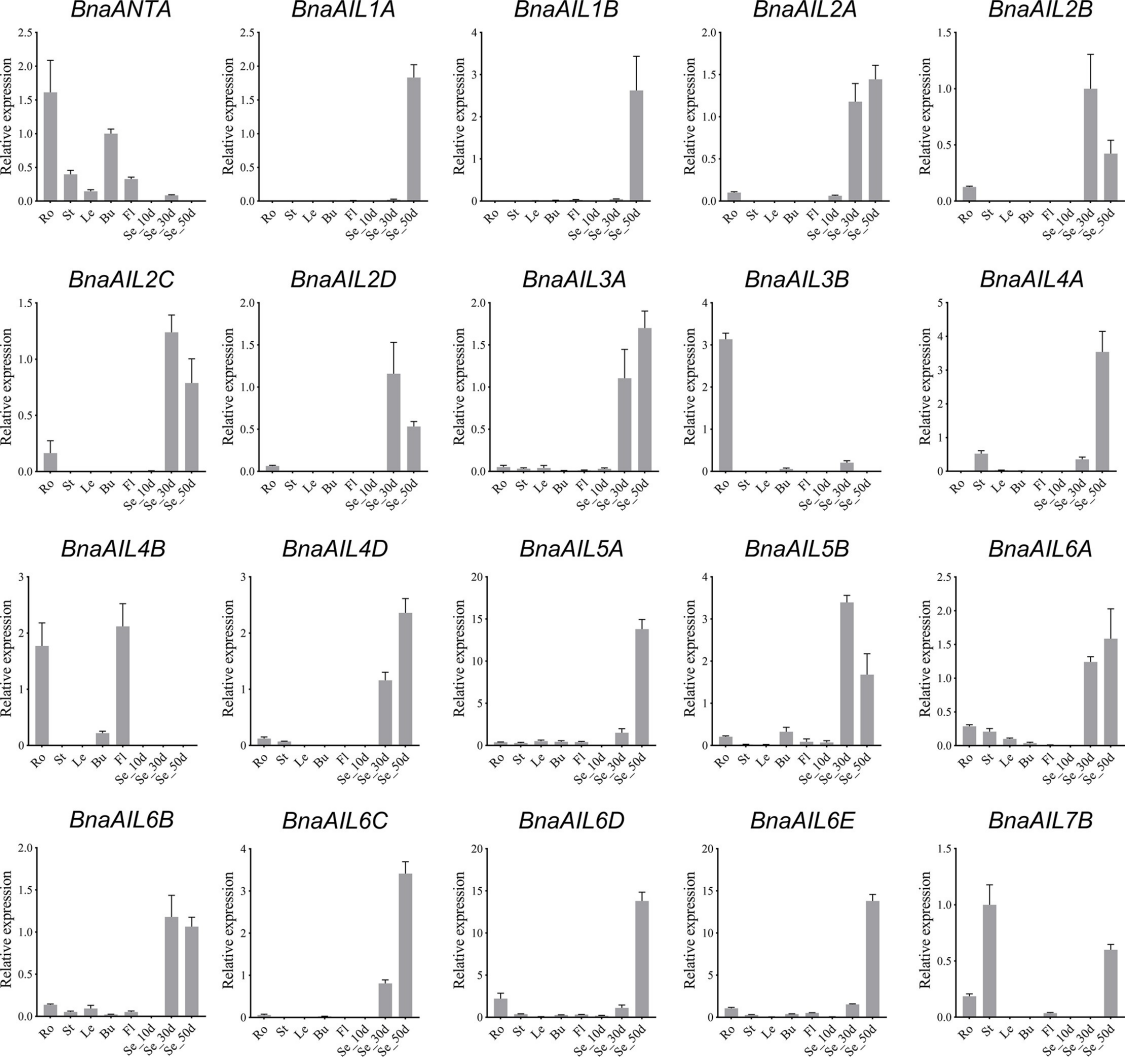
Expression patterns of the *B*. *napus AIL* family genes in different tissues, revealed using qRT-PCR. Ro: root; St: stem; Le: leaf; Bu: bud; Fl: flowers; Se_10d, Se_30d, and Se_50d: seeds 10, 30, and 50 days after flowering. The mean expression values were calculated from three independent replicates. Error bars indicate the standard deviation.

To decipher the physiological functions of the *B*. *napus AIL* family genes, we analyzed the expressions of 20 randomly selected *AIL* genes in eight different *B*. *napus* tissues under normal growth conditions using qRT-PCR (Fig 7). Of these, 17 were more highly expressed in the developing seeds, which is consistent with the fact that the AIL proteins are master regulators of developmental processes, especially during embryogenesis [17, 26, 29]. In addition, AIL proteins are also required for the development of the floral and roots organs [1, 4, 7, 10, 48]. Some *AIL* genes, including *BnaAIL3B*, *BnaAIL4B*, and *BnaANTA*, also showed higher expression levels in the roots and flowers. Furthermore, *BnaAIL7B* was notably highly expressed in the stems. These results further highlight that the *AIL* family genes are involved in the vegetative and reproductive growth in *B*. *napus*, and especially in seed development.

## Discussion

The *AIL* family genes belong to the AP2/ERF superfamily, the members of which are master regulators of plant growth and development, especially of embryogenesis [16, 17, 49]. Furthermore, the cruciferous plants arose from a common ancestor, and have undergone genome duplications and merging during the evolutionary process [50]. Using the eight *A*. *thaliana* AIL protein sequences as a reference, therefore, we identified 99 putatively AIL proteins from various *Brassica* species in this stduy. Among them, 15, 15, and 14 *AIL* family members were identified in the diploid species *B*. *rapa*, *B*. *oleracea*, and *B*. *nigra*, while 26 and 29 *AIL* family members were found in the allotetraploids *B*. *juncea* and *B*. *napus* (Table 1 and S2 Table). More *AIL* family members were identified in the *Brassica* species than in *A*. *thaliana*, suggesting that the *AIL* family genes had undergone a whole-genome triplication among them since their divergence from *A*. *thaliana*, resulting in a significant increase in the numbers of the duplicated genes [50, 51, 52]. Although the allotetraploid *B*. *napus* was formed by the natural hybridization and polyploidization of *B*. *rapa* and *B*. *oleracea* [50], the numbers of *AIL* family genes in these three species were almost equal, indicating that the expansion of the *AIL* family was largely a result of earlier whole-genome and segmental duplications [53]. However, different numbers of *AILs* were identified in the allotetraploid species (*B*. *napus* and *B*. *juncea*) and their parental species (*B*. *rapa*, *B*. *oleracea*, and *B*. *nigra*), suggesting that gene loss or duplication events might have occurred in the *AIL* family genes during the polyploidization of *B*. *napus* and *B*. *juncea*. Additionally, phylogenetic analysis revealed that all the *AIL* family genes could be divided into eight subgroups (Fig 2), which were closely associated with the *AtAIL* groups [17], suggesting that they might share similar functions in the same subgroup.

Previous research revealed that the AIL proteins are members of the AP2 subfamily, part of the AP2/ERF superfamily [16]. Our analysis revealed that two AP2 domains were conserved among all the AIL family proteins, and our sequence comparisons revealed two conserved motifs, referred to as the YRG and RAYD elements, within the AP2 domains (Fig 3 and S1 Fig). These results strongly suggested that the AP2 domain is an important and evolutionarily conserved region necessary for the correct structure or function of the AIL family proteins. The amphipathic α-helices in the RAYD elements were also highly conserved, except in BnaAIL1C, BnaAIL3D, BjuAIL4B, and BnaAIL4D (Fig 3 and S1 Fig), suggesting that these domains might be involved in DNA binding through the interaction of their hydrophobic face with the major groove of DNA [46, 54]. Additionally, the lengths of the AP2-R1 domains were different among the AIL family proteins (Fig 3 and S1 Fig), consistent with previous findings [15, 22], suggesting that they may contribute to differences in the functional specificities of these proteins.

Numerous studies have shown that the AIL proteins were widely involved in the plant growth and developmental processes in young, dividing tissues, including the roots, shoots, floral organs, leaves, and seeds [1, 4, 8, 22, 25, 55]. In the present study, most of the *AIL* genes were expressed at high levels in these tissues, especially in the seeds, embryos, roots, hypocotyls, and cotyledons (Figs 6 and 7), suggesting that these genes may play a role during their development. The expression patterns of some duplicated genes also displayed differences, suggesting that they might have undergone functional divergence after their duplication; for example, *BnaAIL1A* and *BnaAIL1B* were highly expressed in the developing seeds, *BnaAIL1C* was hardly expressed in any of the tissues, and the expression profile of *BnaAIL7A* was completely different to that of *BnaAIL7B* (Fig 6). Additionally, we noticed that *BnaANT* and *BnaAIL6* were expressed in the reproductive tissues (e.g., root, pistil, initial apex, developing seed, embryo, and seed coat), which was consistent with previous results [4, 7, 20, 28], indicating they may play similar roles in *B*. *napus* and *A*. *thaliana*. The expression patterns of these *AIL* family genes in the allotetraploid *B*. *napus* were similar to those observed in its diploid progenitor *B*. *rapa*, which had the higher expression levels in young tissuses [22], suggesting they may play similar roles in both species. In addition, we found that *AIL* genes showed the samilar expression patterns within the same subgroups (Figs 1, 6 and 7), implying that the invariant amino acid residues within the YRG and RAYD elements were controlled by the structure or function of the AP2 domains [46, 47]. Taken together, our results provide the new clues for investigating the roles of AILs in *B*. *napus*.

## Conclusions

In this study, 99 *AIL* family genes were identified from five *Brassica* species, which could be divided into eight subgroups and had closely relationship with the *AtAIL*s. Furthermore, the AIL family genes shared a high similarity among the gene structure, conserved motifs within the same subgroups. The Ka/Ks ratios of orthologous *AIL* gene pairs among *A*. *thaliana* and *Brassica* indicates that the *AIL* genes had undergone strong purifying selection for retention. Additionally, RNA-Seq and qRT-PCR results indicated that the *AIL* family genes might be involved in regulating *B*. *napus* development, especially in the developing seeds. These results enhance the understanding of the evolution and function of *AIL* family genes in *B*. *napus*, providing valuable clues for further research.

## Supporting information

S1 Fig Sequence alignment of all identified AILs from *Arabidopsis* and various *Brassica*. The regions of AP2-R1 to AP2-R2 are shown with blue line. Blue shading represents identical conserved amino acid residues. Color shading represents an α-helix. A detailed description of the two AP2 domains is provided in S3 Table. Bra, *B*. *rapa*; Bol, *B*. *oleracea*; Bni, *B*. *nigra*; Bna, *B*. *napus*; Bju, *B*. *juncea*.
(TIF)

S2 Fig Genome-wide syntenic analysis of all identified *AIL* family genes among *A*. *thaliana*, *B*. *rapa*, *B*. *oleracea*, and *B*. *napus*. The syntenic genes are linked with the red (A subgenome) and light green lines (C subgenome), respectively.
(TIF)

S1 Table Specific primers used to amplify the AIL and reference genes using a qRT-PCR analysis. (XLSX)

S2 Table List of AIL family genes identified from *A*. *thaliana* and *Brassica* species. (XLSX)

S3 Table Details of the AP2 domain in the AIL proteins in *A*. *thaliana* and *Brassica* species. (XLSX)

S4 Table The orthologous *AIL* gene pairs among *A*. *thaliana* and *Brassica* species. (XLSX)

S5 Table *B*. *napus* ZS11 tissues and organs used in this study. (XLSX)
