# Supplementary figures and images for: Genome-wide identification AINTEGUMENTA-like (AIL) genes in Brassica species and expression patterns during reproductive development in Brassica napus L
Source: PLoS One. 2020 Jun 8;15(6):e0234411. doi: 10.1371/journal.pone.0234411 (PMC7279594; doi:10.1371/journal.pone.0234411)

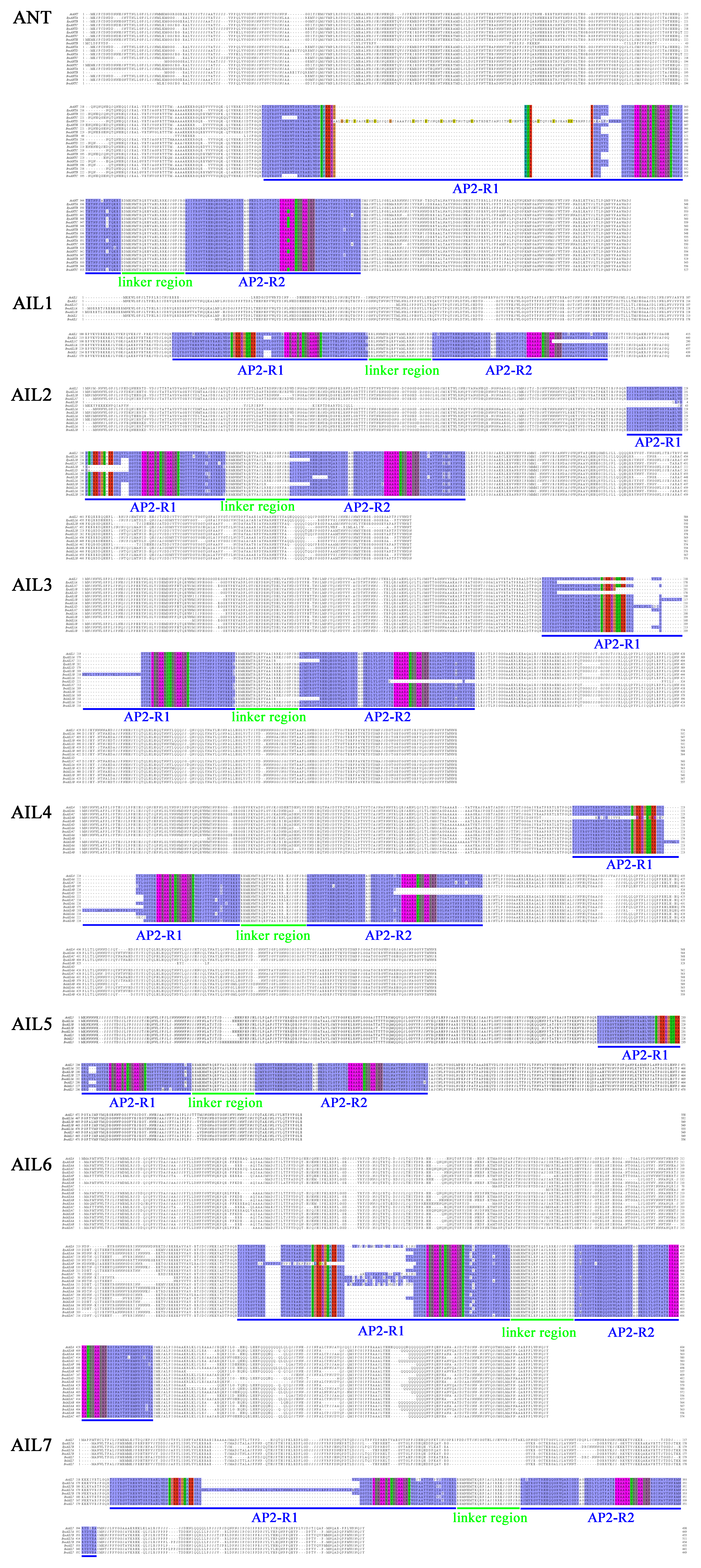

Supplement: S1 Fig — The regions of AP2-R1 to AP2-R2 are shown with blue line. Blue shading represents identical conserved amino acid residues. Color shading represents an α-helix. A detailed description of the two AP2 domains is provided in S3 Table. Bra, B. rapa; Bol, B. oleracea; Bni, B. nigra; Bna, B. napus; Bju, B. juncea. (TIF) [file pone.0234411.s001.tif]

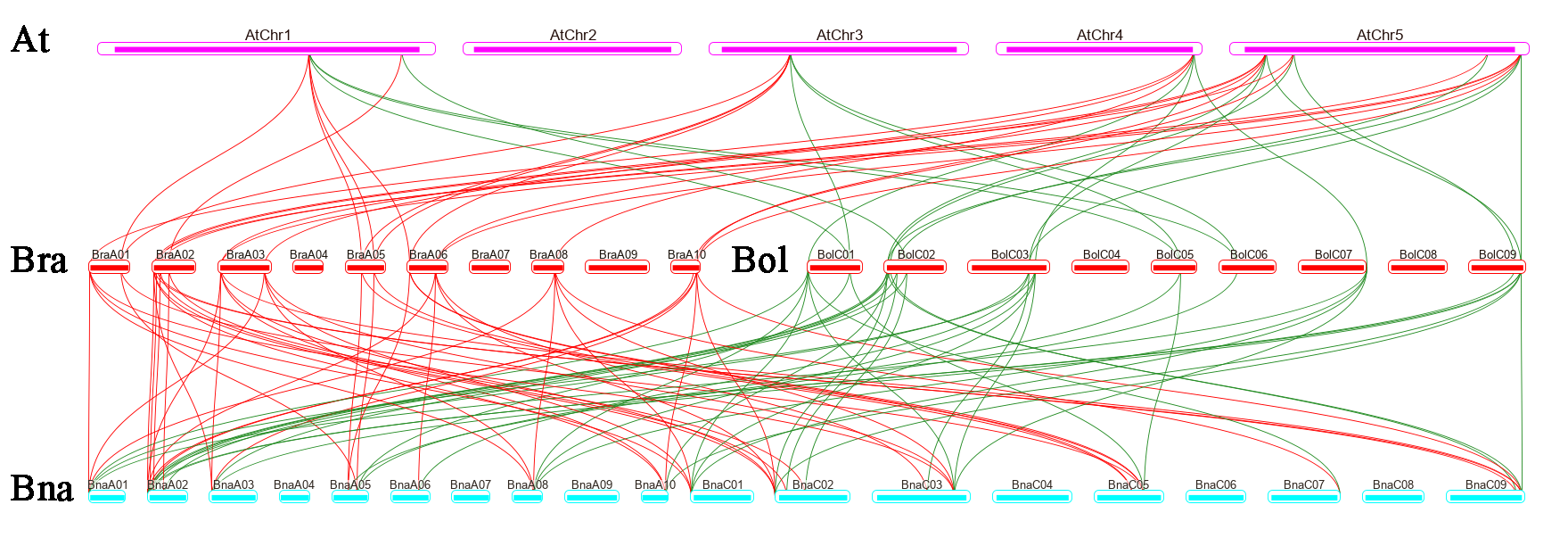

Supplement: S2 Fig — The syntenic genes are linked with the red (A subgenome) and light green lines (C subgenome), respectively. (TIF) [file pone.0234411.s002.tif]
